# Viewpoint: Antimicrobial Resistance Diagnostics Use Accelerator: Qualitative Research on Adherence to Prescriptions

**DOI:** 10.1093/cid/ciad323

**Published:** 2023-07-25

**Authors:** Adélaïde Compaoré, Deborah Ekusai-Sebatta, David Kaawa-Mafigiri, Vida Kukula, Selase Odopey, James Kapisi, Heidi Hopkins, François Kiemde, Halidou Tinto, Rita Baiden, Piero Olliaro, Juvenal Nkeramahame, Sabine Dittrich, Philip Horgan, the ADIP study group, Phyllis Awor, Deborah Ekusai-Sebatta, Heidi Hopkins, David Kaawa-Mafigiri, James Kapisi, Freddy Eric Kitutu, Elizeus Rutebemberwa, Asadu Sserwanga, Alexander Adjei, Rita Baiden, Vida Kukula, Adélaïde Compaoré, François Kiemde, Halidou Tinto, Daniel Valia

**Affiliations:** Clinical Research Unit of Nanoro, Institut de Recherche en Sciences de la Santé, Nanoro, Burkina Faso; Infectious Diseases Research Collaboration; Department of Social Work and Social Administration, School of Social Sciences, Makerere University, Kampala, Uganda; Social Science Department, Dodowa Health Research Centre, Ghana; Social Science Department, Dodowa Health Research Centre, Ghana; Infectious Diseases Research Collaboration; Department of Disease Control, Faculty of Infectious and Tropical Diseases, London School of Hygiene and Tropical Medicine, United Kingdom; Clinical Research Unit of Nanoro, Institut de Recherche en Sciences de la Santé, Nanoro, Burkina Faso; Clinical Research Unit of Nanoro, Institut de Recherche en Sciences de la Santé, Nanoro, Burkina Faso; Social Science Department, Dodowa Health Research Centre, Ghana; Department of Medical Affairs, FIND, Geneva, Switzerland; International Severe Acute Respiratory and Emerging Infection Consortium, Pandemic Sciences Institute, and; Department of Medical Affairs, FIND, Geneva, Switzerland; Department of Medical Affairs, FIND, Geneva, Switzerland; Centre for Tropical Medicine and Global Health, Nuffield Department of Medicine, University of Oxford, United Kingdom; Deggendorf Institute of Technology, European Campus Rottal Inn, Pfarrkirchen, Germany; Department of Medical Affairs, FIND, Geneva, Switzerland; Nuffield Department of Medicine, Big Data Institute, University of Oxford; Department of Medicine, Evidence & Impact - Oxford, United Kingdom

**Keywords:** adherence, prescription, antibiotic, determinants, behaviors

## Abstract

In this Viewpoint, the authors explore the determinants of patients’ prescription adherence behaviors as part of FIND's Advancing Access to Diagnostic Innovation essential for Universal Health Coverage and AMR Prevention (ADIP) trials (ClinicalTrials.gov identifier: NCT04081051). Research findings from Burkina Faso, Ghana, and Uganda show that basic knowledge and understanding of prescription instructions are essential for adherence and can be improved through better communication. However, there are a range of other factors that influence adherence, some of which can be influenced through tailored communication messages from healthcare workers. These messages may contribute to changes in adherence behavior but may require other reinforcing interventions to be effective. Finally, there are some drivers of nonadherence centered around costs and time pressure that require other forms of intervention.

## Teaser

In this Viewpoint, we explore the determinants of patients’ prescription adherence behaviors as part of the Antimicrobial Resistance Diagnostics Use Accelerator clinical trial in Burkina Faso, Ghana, and Uganda.

In this Viewpoint, we combine our experiences exploring the determinants of patients’ prescription adherence behaviors as part of the Advancing Access to Diagnostic Innovation essential for Universal Health Coverage and AMR Prevention (ADIP) trials (ClinicalTrials.gov identifier: NCT04081051). Prescription adherence is defined as obtaining (buying or being given) the prescribed medicine and taking that medicine following the prescription instructions of dosage, frequency, and duration. The ADIP trial was primarily designed to evaluate the impact of a package of interventions on antibiotic prescriptions among patients with acute febrile illnesses at primary health centers in low- and middle-income countries (LMICs). The package of interventions included point-of-care tests, diagnostic algorithms, and training and communication (T&C) packages developed based on preintervention qualitative research [1]. The findings from country-based research were used to develop country-level training and communication interventions based on the assumption that communication messages tailored to the behavioral drivers identified through local research would counter the local drivers of nonadherence and improve patients’ prescription adherence.

This research took place in the clinical trial sites in Burkina Faso, Ghana, and Uganda, and was integrated into the trial intervention and subsequent patient follow-up (for more details, please see the first article in this supplement, by Olliaro et al).

Research findings illustrate a wide range of behavioral drivers that impact patients’ adherence to prescriptions. Basic knowledge and understanding of the prescription instructions (drug type, tolerability, frequency and duration of treatment) are essential for adherence and can be addressed through improved communication. Beyond basic knowledge, research highlights a host of compounding factors that influence adherence, which we hypothesize could be shaped by improved knowledge and persuasion delivered through communication from the healthcare worker (HCW). We believe that the messages will contribute to changes in adherence behavior but would require other reinforcing interventions. Finally, there are some critical behavior drivers centered around costs to patients and time pressure on HCWs, which need other forms of intervention.

## COUNTRY AND RESEARCH SITE CONTEXTS

The Advancing Access to Diagnostic Innovation essential for Universal Health Coverage and AMR Prevention (ADIP) trials took place in the following research sites in Burkina Faso, Ghana, and Uganda:

### Burkina Faso

Nanoro is in the center-west region of the country, approximately 90 km west of the capital, Ouagadougou. Nanoro health district has 28 primary healthcare facilities and 1 rural missionary hospital with an approximate ratio of 6613 inhabitants per health center. In 2020, approximately 64% of the population lives within 5 km, 3.2% within 5–10 km, and 32.9% >10 km away from a primary healthcare facility [2]. The study took place in the villages of Pella and Temnaoré located in the department of Siglé.

### Ghana

Shai-Osudoku District Hospital and St Andrews Hospital hosted the current study. The research sites are located in the Shai-Osudoku District of the Greater Accra Region of Ghana. Shai-Osudoku District is a periurban district with Dodowa as its capital. The study involved healthcare providers and patients/caregivers/household decision makers within the Shai-Osudoku District.

### Uganda

In Uganda, research was conducted at 3 sites: Aduku (Kwania district, northern Uganda), Kihiihi (Kanungu district, southwestern Uganda), and Nagongera (Tororo district, eastern Uganda) Health Centers IV. These public health facilities serve a mostly rural catchment area across 3 regions in the country. They are the main public health facilities and together with private for-profit facilities contribute to the alternatives available for care. The target populations are of low socioeconomic status and characterized as largely peasant farming communities in the catchment areas [3].

## FACTORS THAT HINDER PRESCRIPTION ADHERENCE

### Knowledge and Understanding of Basic Prescription Instructions

Communication from the HCW is crucial in conveying basic instructions about which drug has been prescribed and is to be obtained as well as the frequency and duration of the course.

There are many possible reasons for ineffective communication that leaves the patient confused. Consequently, patients may take medicine with an incorrect dose, duration, or frequency, when parts of the prescription instructions are missing from the dialogue because the HCW cannot communicate in a language that the patient understands well or because the patient is elderly, has a low education level or is illiterate, or has difficulty comprehending what is spoken to them.

Attitudes and manners of HCWs, patients, and carers impact the ability to successfully communicate and influence a 2-way dialogue allowing questions, and clarity, from the patient. Noticeably in Burkina Faso, patients arrive to consultations stressed. Attitudes and approaches of the HCW are essential to reduce this stress to ensure that the prescription information is taken on board.

Communication is supported when both HCWs’ and patients’ attitudes are respectful and positive, when information is communicated using pictures and images, when there is a caregiver or family support person present to hear the prescription instructions, when information is written down in a way that can be referred to later, and when an appropriate language is used.

The training and communication packages developed in the study introduced consistency in communication messages and practices to address these factors.

The amount of time the HCWs have for consultation and communication is essential, as is the division of roles between HCWs and pharmacists in health centers (see section ‘High workload, short consultation time, and impact on communication’ below). Outside of a trial setting, it would seem that investment to reduce high HCW workload and expand available time for consultation will be needed to realize these benefits.

### Compounding Behavioral Drivers

Beyond knowledge of basic prescription instructions, the research highlighted compounding behavioral drivers that hinder patients’ prescription adherence. These are drivers of routine and habit, social norms, and the influence of others. We consider that behavioral drivers could be influenced by knowledge and persuasive communication from the healthcare provider to the patient during prescribing. However, we appreciate that these are unlikely to change deep-seated behaviors and beliefs independently, and complementary interventions are likely needed.

The research findings indicate that many patients stop taking prescribed medicine when they feel better (which, given cost issues described below, may be influenced by a desire to save medicine for later for themselves or others). This is driven by social norms, routines, habits, and a lack of knowledge of the health consequences of their actions. Similarly, driven by habits and social norms, patients halt medication when they experience side effects. However, medication is stopped without understanding which side effects are medically acceptable (meaning they should continue with the drug) and which are more serious (when they should seek further care).

#### Patients Do Not Take Medicines at the Right Time of Day or Miss Dosages

Forgetfulness is often cited as the reason why the medicine is not taken at the prescribed time. Aids are required, including linking the time of day to take the medication with mealtimes, the position of the sun, or other daily events that happen in the community such as prayer calls/time; written or pictorial instructions for the patient to take home; support from caregivers or family members to give reminders; and the use of watches or phones to set alarms.

Patients report that work or school schedules hinder them from taking medicines at the required times. It is implied that patients consider that they need to be at home to take the medication, which affects minors who require medication while at school/during the school day and thus away from their usual caretaker/parent.

#### Influence of Others and Preconceived Beliefs

Patients’ preconceived beliefs and common religious and cultural practices combine with influence from community leaders to hinder prescription adherence. Religious leaders may advocate for prayer instead of medicine, traditional healers may advocate for the use of traditional medicines, and community members who have, for example, a status of advisor on child health (Burkina Faso) may negatively influence patients’ adherence to the prescription. Communication messages try and counter the effects of these alternative treatments or advise simultaneous use.

#### Family Dynamics

In Burkina Faso, participants report the need for parents to think strategically to ensure their child takes the medicine, prepare special food to take with the medicine, and flatter the child. At the same time, it is noted that the role of the father, “as a policeman,” is important to ensure the drug is taken according to the prescription instructions.

#### Support From Others and Handing Over Responsibility

Adherence is reportedly improved when the patient is accompanied to the health center by a caregiver or family member, who also hears the instructions and adherence messages. Difficulties are noted when no one is providing support or a change in the person giving support once back at home, where the caretaker (eg, a parent) who attended the clinic is away for work during the course of the day and another family member has to take care of the patient (eg, in Uganda).

However, social support can hinder adherence. Community members reported sharing their drugs with a family member exhibiting similar symptoms for which they were being treated and for whom they may not find medicines. At times, patients hand over responsibility for ensuring their child takes medication to an alternative caregiver when they have an important community obligation, such as attending a funeral, sometimes lasting days away from home (Uganda). However, this person may not have heard the prescription instructions, and instructions may or may not be passed on and followed. Involving an alternative caregiver in the treatment administration can be problematic in terms of adherence (Burkina Faso).

#### Blunt Messages and Fear Used in Support of Prescription Adherence

In Ghana, HCWs, at times, use very blunt messages, discussing the severity of the patient's illness and instilling fear to scare patients into following prescription adherence. Reference to laboratory results reinforces the seriousness of the situation and the severity of the patient’s condition.

#### Evidence of Effective Persuasion

In the follow-up interviews with patients in the trial, participants responded positively to the communication messages of the T&C interventions.

However, analysis of quantitative data on prescription adherence collected in the study showed different effects on adherence to antibiotic prescriptions between patients given the T&C intervention and those not given the intervention. In Uganda, adherence improved (42.5% vs 71.3%), whereas it remained largely the same in Burkina Faso (78.2% vs 78.1%) and dropped in Ghana (90.3% vs 82.9). It is still being determined if this is an accurate reflection of the inability of the communication messages to improve adherence or if biases in either reporting of adherence or biases through the nature of a clinical trial have skewed this quantitative analysis. Thus, while we believe all the qualitative evidence points to the benefit of effective communication between HCWs and patients to support adherence to the prescription, we do not have quantitative evidence demonstrating this through this clinical trial.

## COST AND TIME DRIVERS

### Cost Burden to Patients, Drug Stockouts, and Impact on Nonadherence

Within poor communities, the economic burden of healthcare influences patients’ adherence to prescriptions across the 3 countries and locations studied.

In Ghana, the national insurance scheme covers the costs of some medicines from health facilities for those in the scheme. In Burkina Faso, free healthcare is provided to pregnant women and children <5 years old; in theory, providing cheap drugs at healthcare facilities and support schemes should alleviate the economic burden of purchasing medication in these population groups. In Uganda, free care, including a few tests and essential drugs, are provided to all patients by the government. However, in practice, this does not appear to be the case. Due to drug and test stockouts, many patients miss these free services, and cost burdens impact patients’ adherence to prescriptions.

While national schemes exist, they are, by their nature, limited. In Ghana, the national insurance scheme does not cover all medicines in health facility pharmacies, nor most medicines obtained at private pharmacies/drug shops. In Burkina Faso, the scheme is limited to medications received from the health facility pharmacy and for pregnant women and children aged <5 years. Whether these schemes cover a patient or not, medicines may not be available at the health facility pharmacy because the prescribed medications are not routinely stocked there or because drug stockouts mean the drugs are unavailable when needed.

Prescribed medicines not available at the health facility must be purchased elsewhere. Drugs purchased at private drug shops are costly and more expensive than drugs that can be bought at public health facilities. In addition, especially in rural settings, patients must pay for transport, such as motorcycle taxis, to reach the alternative drug shops. Additional costs put further pressure on already stretched household finances, and families must choose between competing healthcare needs.

Patients use a variety of strategies to try and overcome unaffordability problems. They may find the money needed to buy the prescribed medicine in full—by selling cereals, poultry, or other assets or borrowing money (Burkina Faso). The time required to raise funds has the effect of starting a dose later than intended (Burkina Faso, Uganda). Patients may act to change the prescribed medicine so that it is cheaper by asking the HCW for help—generally reducing the number of prescribed drugs, prescribing more affordable drugs (Burkina Faso), or acting to reduce the cost of the medicines by delaying the start of doses, suspending or stopping treatment early (Uganda), buying alternative drugs or herbal medicines that are cheaper (Burkina Faso), or simply not buying the prescribed medication (Ghana).

Once patients visit private drug shops, further factors impact prescription adherence. Patients can be persuaded to buy less medicine than prescribed or to buy something else. Moreover, there is a sense (from HCWs) that while pharmacies in health facilities will generally provide prescription instructions, there is little control over the prescription instructions provided by private drug shops.

While drug stockouts are highlighted as a significant factor in increasing the cost to patients and contributing to a lack of adherence, unaffordability is also reportedly driven by late presentation at health facilities, leading to more expensive treatment such as intravenous injections (Burkina Faso), and wrong and multiple diagnoses, resulting in the need to purchase numerous medicines (Burkina Faso).

While financial constraints are reported in isolation from other factors, it is anticipated that issues of finance interact with the beliefs of the patient and household decision-maker about the duration of medicine needed for the patient to recover. For example, household heads report that they would not purchase a full course of treatment if the patient showed signs of improvement following a small initial dose of the prescribed medicines (Uganda).

### High Workload, Short Consultation Time, and Impact on Communication

Time pressures are, perhaps not surprisingly, reported as a significant factor in HCWs’ ability to effectively communicate prescription instructions to the patient and impact prescription adherence.

Outside of the intervention arm of the trial, HCWs report long queues of patients needing consultation and constant pressure to get through the line of patients during the day. With too little time for doctors to adequately consult patients, communication of prescription instructions takes a second priority, can be done poorly, or is passed on as the pharmacist's role. While this division of responsibilities may seem practical to free up the HCW’s time, it has drawbacks. To date, communication from pharmacists is limited to the basic prescription information (and would therefore need to be complemented with persuasive messages). Compounded with issues of the unavailability of prescribed medicines at health center pharmacies, either because this is not a criterion for prescribing practices or because of stockouts as described above, this would result in many patients missing out on reliable and persuasive messaging.

## CONCLUSIONS

Reasons why patients do not adhere to prescriptions are many and varied. Some reasons center around the ability of HCWs to effectively communicate the basic prescription instructions to the patient and for the patient to understand these instructions. Beyond this, various social and cultural factors pull patients away from adherence, which we consider may be influenced by communication messages but will likely also need other reinforcing interventions. However, some fundamental problems such as the financial pressures faced by poor communities, and an overstretched health system, will require deeper and wider-reaching solutions.

Existing systematic reviews exploring behavior drivers for medicine adherence across diseases highlight the limited number of studies conducted in LMICs. In their review of articles published between 1970 and 2005, Jin et al [4] note that “few studies on compliance have been performed in Asian and developing countries where most of the world's population resides,” while Chauke et al [5] note in their review of studies published from 2008 to 2018 that they could identify only 6 eligible studies out of 154 screened that “presented evidence on factors influencing poor medication adherence amongst patients in low- and middle-income countries.” Similarly, Schmiege et al [6] note that “findings are biased towards higher-income countries and Western countries, highlighting that more evidence is needed from lower- and middle-income countries and other regions.”

According to Jin et al, “More studies on factors influencing compliance in these countries or regions would be helpful to fill in the knowledge gap and contribute to formulating international strategies for countering non-compliance” [4].

Within the available literature, overall reflecting high-income and upper-middle-income countries, we see a commonality in behaviors identified in this study, with determinants such as costs and income, patient knowledge, and difficulty in getting prescriptions filled [6]. What is not so easily seen in these systematic reviews is the importance of cultural and interpersonal drivers, such as the influence of religious or traditional leaders and habitual use of traditional medicines, or the dynamics and roles within the family, such as transfer of responsibility (but without transfer of knowledge) to other family members and the “policeman” role of fathers.

This study highlights the complex interwoven web of factors, including cultural factors, that interact with each other and push and pull on the patient/caregiver adherence behavior. We suggested that an in-depth understanding of the interrelation of these factors is needed to design effective interventions.

The discussion above focused on identifying behavioral drivers that were used to develop a T&C intervention in support of prescription adherence. However, as the findings demonstrate, other additional forms of intervention will be required. In the second phase of the study we investigated an intervention design to address prioritized behavior drivers of nonadherence, using behavioral frameworks and a modified version of the behavior change wheel [7]. This will be published shortly.

We consider that the behavioral drivers described above, and summarized in the Supplementary Material, are the most important and are broadly common across the 3 countries and research sites, with some nuance difference between countries. In each country, additional behavioral drivers were identified (and are described in the individual research manuscripts in this supplement). While these commonalities were found, the value of country-specific research and communication messages was confirmed to address specific issues.

## Supplementary Data

Supplementary materials are available at *Clinical Infectious Diseases* online. Consisting of data provided by the authors to benefit the reader, the posted materials are not copyedited and are the sole responsibility of the authors, so questions or comments should be addressed to the corresponding author.

## Supplementary Material

ciad323_Supplementary_Data

## Notes

***Author Contributions.*** All authors were involved in the drafting and revising of the manuscript and approved the final version for submission. Editorial assistance in the preparation of the manuscript was provided by Stuart Wakelin and funded by FIND.

***Acknowledgments.*** The overarching protocol for the multicountry clinical trial, with nested qualitative components (part of which is commented on in this Viewpoint), was reviewed and approved by the Human Research Ethics Committee of the University of Oxford, United Kingdom. In addition, country-specific protocols were reviewed and approved by relevant regulatory authorities and national and/or institutional ethics committees in all participating countries.

***Advancing access to Diagnostic Innovation essential for UHC and AMR Prevention (ADIP) Study Group*.** Uganda: Phyllis Awor, Deborah Ekusai-Sebatta, Heidi Hopkins, David Kaawa-Mafigiri, James Kapisi, Freddy Eric Kitutu, Elizeus Rutebemberwa, and Asadu Sserwanga. Ghana: Alexander Adjei, Rita Baiden, and Vida Kukula. Burkina Faso: Adélaïde Compaoré, François Kiemde, Halidou Tinto, and Daniel Valia.

***Financial support*.** This part of the study was funded by the Swiss Agency for Development and Cooperation (SDC), the Federal Ministry of Economic Cooperation and Development (BMZ), and the UK Department For International Development, now the Foreign, Commonwealth and Development Office (FCDO).

***Supplement sponsorship.*** This article appears as part of the supplement “Using Diagnostic Tools to Support Antimicrobial Stewardship and Improve Outcome in Resource-Limited Contexts,” sponsored by FIND.
