# Supplementary material for: Viewpoint: Antimicrobial Resistance Diagnostics Use Accelerator: Qualitative Research on Adherence to Prescriptions
Source: Clin Infect Dis. 2023 Jul 25;77(Suppl 2):S206–10. doi: 10.1093/cid/ciad323 (PMC10368411; doi:10.1093/cid/ciad323)
Supplement: ciad323_Supplementary_Data [file ciad323_supplementary_data.docx]

# **Supplementary information 1**

# **Summary of behavioral drivers common across countries with additional country-specific perspectives**

| **Topic** | **Drivers of prescription adherence/ non-adherence**  **All countries** | **Country specific perspectives** |
| --- | --- | --- |
| Knowledge and understanding of basic prescription instructions    Two way dialogue allowing questions and clarity from the patient | Language barriers, the HCW cannot communicate in a language that the patient understands well. |  |
|  | Elderly, low education level, illiterate, or other reasons that makes comprehension of messages difficult |  |
|  | Attitudes and manners of HCWs and patients and carers  Communication supported with attitudes are respectful and positive | Patients arrive at consultations stressed (Burkina Faso) |
|  | Communication using pictures and images |  |
|  | Patient is accompanied by a caregiver or family support person who also hears the prescription instruction |  |
|  | Prescription instructions are written in a way that can be referred to later, in an appropriate language |  |
| Patients stop taking medicines when they feel better or experience side effects | Routine and habits  Social norms  Influence of others  Lack of knowledge of health consequences of actions; lack of knowledge to differentiate between medically acceptable side effects and when further care is needed.  May be linked to patient financial constraints |  |
| Patients do not take the medicines at the right time of the day or miss dosages | Forgetfulness. Aids support forgetfulness |  |
|  | Work or school schedules hinder patients taking medicines (implied that medicines should be taken at home) |  |
| Influence of others, preconceived beliefs | Religious leaders advocate for prayer instead of medicines; traditional healers advocate for use of traditional medicines. | Community members with informal status of child health advisor negatively influence patients adherence to prescription (Burkina Faso) |
| Family dynamics |  | Children dislike taking medicine. Parents prepare special food to take with medicine and flatter the child. Fathers acts as a ‘policeman’ to ensure the child take medicine |
| Support from others and handing over responsibility | Adherence improved when a caregiver or supporter accompanies the patient, hears the prescription instructions and can support the patient to follow. | Difficulties arise when responsibility is passed onto another family member or caregiver who didn’t attend the clinic and hear the prescription instructions. (General finding Burkina Faso)  For example when the parent who attended the clinic with the patient is at work during the day and another family member is asked to give the medicines to the patient. Or for example if the parent is away to attend a multi-day funeral. (Uganda) |
| Sharing of medication | Sharing of medication with family members with similar symptoms, due to concern that medicines for the additional family member might not be found. |  |
| Blunt messaging |  | HCWs in Ghana may instil fear in the patient using very blunt messages to scare them into following the prescription |
| Costs and time | Free healthcare and medicines available through national health schemes often do not materialise due to drug and test stockouts, combined with patient financial constraints reduces the ability of patients to obtain the prescribed medicines. | (Ghana) National insurance scheme does not cover all medicines in health facility pharmacies nor private pharmacies/drug stores.  (Burkina Faso) Free health care is in theory provided to pregnant women and children under five, however the scheme is limited to medicines received from the health facility pharmacy. However prescribed medicines may not be routinely stocked at the health facility or drug-stockouts mean the drugs are unavailable when needed. |
|  | Frequent stockouts cause prescribed medicines to become unavailable at health facility pharmacies, meaning that they need to be purchased from other locations. | Stock outs at |
|  | Drugs purchased at private drug shops are more expensive than those purchased at public health facilities, and patients have additional costs to pay such as transport to reach alternative shops, adding further pressure to household budgets. |  |
|  | The time required to raise money by financially constrained households can delay the start of the prescription. | For example, in Burkina Faso patients may need to sell cereals, poultry or other assets of borrow money. |
|  | Financial constraints – coping mechanisms | To cope with the cost of the prescribed medicines patients may ask the HCW to reduce the number of prescribed drugs or to prescribe more affordable dugs, may buy alternative medicines or herbal medicines which are cheaper (Burkina Faso).  In Uganda, patients may delay the start of the dose, suspend or stop treatment early to reduce costs. In Ghana patients may simply not buy the prescribed medicine |
|  | Consequences of visiting drug shops include persuasion from the drug seller to buy a different drug, or a different or part quantity, and little confidence by HCWs in the messages accompanying the medication. |  |
|  | Late presentation at clinics reportedly leads to a need for more expensive medication or formulations. | For example in Burkina Faso, it is reported that more expensive treatment such as intravenous injections are given after late presentation.  And in Burkina Faso incorrect and multiple diagnosis increase the number of drugs and associated costs for patients. |
| High workloads, short consultation time | High workloads and short consultation time impact on the ability of the HCW to communicate to the patient and caregiver. |  |
